# Supplementary material for: Resilience and Positive Wellbeing Experienced by 5–12-Year-Old Children with Refugee Backgrounds in Australia: The Childhood Resilience Study
Source: Int J Environ Res Public Health. 2024 May 15;21(5):627. doi: 10.3390/ijerph21050627 (PMC11121680; doi:10.3390/ijerph21050627)
Supplement: Supplementary file 1 [file ijerph-21-00627-s001.zip › ijerph-2973516-supplementary.pdf]

International Journal of
Environmental Research and Public Health

MDPI

Table S1. Mean CRQ-P/C domain and scale scores by gender for children of refugee background, with Tobit logistic regression modelling differences for boys compared to girls (n=102).

DOMAIN	Items (range[1])	Girls (n=49)	Boys (n=53)	Tobit Regression (comparing boys with girls)	
CRQ Scale		Mean [95%CI]	Mean [95%CI]	β [95%CI]	p-value
**PERSONAL strengths**					
Positive self	4 (0 - 16)	13.2 [12.4 - 13.9]	12.9 [12.2 - 13.5]	-0.4 [-1.6 - 0.7]	0.435
Positive future	4 (0 - 16)	13.1 [12.4 - 13.9]	13.4 [12.6 - 14.1]	0.4 [-1.1 - 1.9]	0.624
Emotion regulation	3 (0 -12)	8.1 [7.3 - 8.8]	8.2 [7.6 - 8.7]	0.1 [-0.9 - 1.1]	0.869
Mean personal domain score	11 (0 - 16)	11.5 [10.9 - 12.2]	11.5 [11.0 - 12.0]	0.1 [-1.1 - 1.2]	0.927
**FAMILY strengths**					
Connectedness	4 (0 - 16)	13.4 [12.7 - 14.1]	12.9 [12.1 - 13.7]	-0.6 [-2.0 - 0.9]	0.458
Basic needs	4 (0 - 16)	12.7 [12.0 - 13.5]	13.5 [12.8 - 14.1]	0.9 [-0.3 - 2.2]	0.153
Guidance	3 (0 - 12)	8.5 [7.9 - 9.2]	8.3 [7.6 - 9.0]	-0.2 [-1.3 - 0.8]	0.656
Mean family domain score	11 (0 - 16)	11.6 [11.0 - 12.2]	11.6 [11.0 - 12.2]	0.0 [-1.1 - 1.2]	0.958
**SCHOOL strengths**					
Teacher support	4 (0 - 16)	13.1 [12.4 - 13.8]	12.4 [11.6 - 13.1]	-1.1 [-2.6 - 0.4]	0.133
School engagement	4 (0 - 16)	13.7 [13.0 - 14.3]	13.4 [12.9 - 14.0]	-0.3 [-1.3 - 0.8]	0.625
Friends	3 (0 - 12)	9.2 [8.6 - 9.8]	8.9 [8.3 - 9.5]	-0.3 [-1.3 - 0.6]	0.508
Mean school domain score	11 (0 - 16)	12.1 [11.6 - 12.6]	11.7 [11.2 - 12.2]	-0.9 [-1.9 - 0.1]	0.082
**COMMUNITY strengths**					
Cultural connectedness	4 (0 - 16)	12.7 [11.9 - 13.4]	12.6 [11.9 - 13.3]	-0.0 [-1.2 - 1.2]	0.990
Connectedness to language	4 (0 - 8)	7.0 [6.6 - 7.3]	6.6 [6.1 - 7.1]	-0.3 [-0.9 - 0.3]	0.355
Mean community domain score	8 (0 - 16)	9.7 [9.3 - 10.2]	9.2 [8.6 - 9.9]	-0.5 [-1.3 - 0.4]	0.297
**Total RESILIENCE score**					
Mean total scale score	43 (0 - 16)	11.4 [10.9 - 11.8]	11.2 [10.8 - 11.6]	-0.2 [-1.0 - 0.6]	0.614

[1] Response options ranged from 0 'Not at all' to 4 'All of the time", with exception of language where response options ranged from 0 'Not at all' to 2 'A lot".
